# Effects of Coix Seed Extract, *Bifidobacterium* BPL1, and Their Combination on the Glycolipid Metabolism in Obese Mice

**DOI:** 10.3389/fnut.2022.939423

**Published:** 2022-07-18

**Authors:** Wei Zhang, Xiuzhen Jia, Yuhan Xu, Qiaoling Xie, Meizhen Zhu, Hesong Zhang, Zifu Zhao, Jingyu Hao, Haoqiu Li, Jinrui Du, Yan Liu, Wei-Hsien Liu, Xia Ma, Weilian Hung, Haotian Feng, Hongwei Li

**Affiliations:** ^1^School of Public Health, Xiamen University, Xiamen, China; ^2^Inner Mongolia Dairy Technology Research Institute Co., Ltd., Hohhot, China; ^3^Yili Innovation Center, Inner Mongolia Yili Industrial Group Co., Ltd., Hohhot, China

**Keywords:** *Coix* seed extract, *Bifidobacterium* BPL1, obesity, inflammation, glycolipid metabolism, gut flora

## Abstract

Coix seed extract (CSE) and probiotics have been reported to regulate glycolipid metabolism *via* different modes of action. We tested the effects of CSE, *Bifidobacterium* BPL1, and their combination to determine their effects on glycolipid metabolism in obese mice. Male C57BL/6J mice were fed a high-fat diet for 8 weeks to establish an obesity model. Obese mice were selected and divided into four groups: the model control group and three intervention groups. After 10 weeks of continuous gavage intervention, the mice in the intervention groups exhibited lower body weight (lower about 2.31 g, vs. HFD mice 42.23 g) and epididymal (lower about 0.37 g, vs. HFD mice 2.5 g) and perirenal fat content (lower about 0.47 g, vs. HFD mice 0.884 g); decreased fasting blood glucose, total cholesterol, triglycerides, and VLDL; and increased HLDL, respiratory exchange ratio, energy expenditure, and amount of exercise performed. CSE, BPL1 and their combination can effectively control the weight gain in obese mice, reduce fat content, and regulate blood lipids and abnormal blood sugar. These results may be related to reduce the chronic inflammatory states, improve energy metabolism, exercise, relieve insulin sensitivity, and reduce lipid synthesis *via* the intervention of CSE, BPL1 and their combination. Compared with the single use of CSE alone, the combination of CSE + BPL1 can better exert the regulation function of intestinal flora, and change in the abundance of bacteria that could improve the level of inflammatory factors, such as increasing *Bifidobacterium*, reducing *Lactococcus*. Compared with the use of BPL1 alone, the combination of CSE and BPL1 can better regulate pancreatic islet and improve blood sugar. CSE may act directly on body tissues to exert anti-inflammatory effects. BPL1 and CSE + BPL1 may improve the structure and function of the intestinal flora, and reduce tissue inflammation.

## Introduction

Obesity and obesity-related metabolic diseases have becomegrowing global public health problems. According to the World Health Organization, overweight and obesity result in 2.8 million deaths and 35.8 million disability-adjusted life years (DALYs) each year, accounting for approximately1 2.3% of DALYs; furthermore, globally 1/5 of adults are expected to experience obesity (1). The shift in modern dietary patterns toward energy-dense diets rich in fats and/or sugars is strongly associated with overweight and metabolic syndrome. However, few diet pills are available on the market, and these have many side effects, making them unsuitable for long-term use (2). Therefore, exploring more effective and safe anti-obesity dietary supplements from natural substances is necessary to prevent or treat obesity and obesity-related metabolic diseases. x *Coix lacryma-jobi* L. var. *ma-yuen* (Roman.) Stapf is a plant species belonging to the Poaceae family, whose dried and mature seed kernels are edible and have medicinal properties (3). The main active components of *Coix* Seeds are esters, unsaturated fatty acids, carbohydrate and lactams (4, 5). Body constitution in traditional Chinese medicine (TMC) is believed be a relatively stable inherent trait formed under the combined action of congenital heredity and acquired environment, and is the background and foreground factor for the occurrence of diseases (6). TCM constitution is related to obesity, and different constitution types have different chances of developing obesity. Existing research shows that Phlegm-dampness type, Qi-deficiency type, and phlegm-dampness + blood stasis types were significantly correlated with overweight (6–9). However, TMC believes that Coix seed has the diuretic functions and swelling, spleen and dampness, blood circulation and stasis (10, 11), which may improve obesity-related body constitution. Recent pharmacological studies have shown that the polysaccharides, proteins, fatty acids, and polyphenols of *Coix* seeds have pharmacological effects such as regulating glucose and lipid metabolism and intestinal microbiota (3–5).

Similarly, *Bifidobacterium* is widely used as a food supplement in dairy products. Studies have found that it can affect the absorption of dietary fat and fat-soluble vitamins by regulating intestinal metabolites, promoting lipid absorption, maintaining intestinal barrier function, and reducing inflammation levels. Probiotics can also improve gut dysbiosis and are beneficial in preventing or treating obesity (12, 13). Endocrine signaling regulates triglyceride, cholesterol, glucose and energy homeostasis (14, 15). Human trial experiments have found that *Bifidobacterium* animalis subsp. Lactis (BPL1) CECT 8145, as viable cells. In heat-killed form, it reduces anthropometric adiposity biomarkers in abdominally obese individuals (16, 17). Animal experiments found it could regulate lipid metabolism and antioxidant responses (18, 19).

Both *Coix* seed extract (CSE) and probiotics can regulate glycolipid metabolism and the intestinal microbiota. Therefore, this aimed to integrate traditional Chinese and Western medicine to nourish from the inside and help in weight management. It determined the effects of CSE, BPL1 and their combination on weight loss, lipid levels reduction, and blood glucose levels regulation in high-fat diet-induced obese mice. We also explored the potential mechanisms behind these effects.

## Materials and Methods

### Source of Materials

CSE was provided by Beijing Yili Technology Development Co., Ltd., (Beijing, China), and the recommended dose for humans was 1 g/d (20). Preparation of CSE: The plant material was Coix lacryma-jobi L. var. mayuen (Roman), using water and ethanol extraction, and the extraction ratio is 1:10. The procedure was that 1.2 kg dry powder and ground coix seed extract were extracted with 95% ethanol reflux for 2 h, the extracts were combined in a rotary evaporator and concentrated, then the extract was dried in a vacuum drying oven at 80°C to obtain 102.3 g of CSE (21), which was frozen at −4°C. The active ingredients are coix seed fat, protein, carbohydrates, etc. The CSE used in this experiment contains > 30% polysaccharides. For the product inspection sheet, see Supplementary Material 3 Coix Seed Product Inspection Sheet. For *Bifidobacterium* animalis subsp. Lactis (BPL1, CECT8145, 10^10^ colony forming units) were obtained from Archer Daniels Midland Co-Biópolis (Valencia, Spain), and the recommended dose for humans was 1×10^10^*cfu*/d (16). The recipe groups are listed in Table 1.

**TABLE 1:** Animal experimental protocol.

Group	Test substance	Recommended dose for humans [g/d or cfu/d]	Intervention dose in humans [mg/d.kg or cfu/d.kg] *	Intervention dose in mice [mg/d.kg or cfu/d.kg]	Gavage concentration [mg/ml or cfu/ml]
ND	Normal saline	–	–	–	–
HFD	Normal saline	–	–	–	–
Group 1	CSE	1.00	16.67	166.67	16.67
Group 2	BPL1	10^10^	1.67×10^8^	1.67×10^9^	1.67×10^8^
Group 3	CSE	0.50	8.33	83.33	8.33
	BPL1	10^10^	1.67×10^8^	1.67×10^9^	1.67×10^8^

*Calculating intervention dose in humans, we set the adult standard weight was 60 Kg (69).

### Ethical Approval Statement

All experimental procedures were performed in accordance with the guidelines of the Institutional Animal Care and Use Committee of the Laboratory Animal Center of Xiamen University and the International Association of Veterinary Editors guidelines for the Care and Use of Laboratory Animals. The protocols for animal use were reviewed and approved by the Animal Ethical and Welfare Committee of the Laboratory Animal Center of Xiamen University (Approval No. XMULAC20200185).

### Animal Experiments

C57BL/6J male mice (*n* = 150) with an average body weight of 19.02 ± 0.88 g were purchased from Shanghai Slack Laboratory Animal Co., Ltd. They were reared at 22°C, 10–60% humidity, 12 h/12 h light-dark cycle, and free access to water. After 1 week of adaptive feeding, 12 mice were selected by using random number method and continued to be fed with a normal diet (ND) [Beijing Keao Xieli Feed Co., Ltd.; Beijing Feed Certificate (2018) 0673] for 8 weeks. The remaining mice were fed a high-fat diet (Research Diets, Inc., D12492), in which fat accounts for 60% of the heat energy, for the same duration to establish mouse models of obesity. High-fat feed was purchased from the Jiangsu Shuangshi Laboratory Animal Feed Department [Su Feed Certificate (2017) 05005]. After the feed, mice with a 40% increase in body weight were considered successful obesity models (22) and were randomly divided into one model control (HFD) and four intervention groups, with 12 mice in each group.

In the CSE and BPL1 alone intervention groups, the mice were administered CSE and BPL1 doses 10 times the recommended human doses (23) (Table 1). In the ND and HFD groups, the mice were administered normal saline *via* gavage. The gavage volume was set at 0.1 ml/10 g mouse body weight. The mice’s body weight and feed intake per cage were measured weekly, and energy intake was calculated based on food intake during the 10-week intervention period. Fasting blood glucose (FBG) levels were measured at weeks 0, 4, 8, and 10. At the end of the intervention, eight mice from each group were randomly selected for oral glucose tolerance tests (OGTT). After 12-h fasting, the mice were immediately administered a 20% glucose solution by gavage according to their body weight (10 μl/g). Blood was drawn from the tail vein of the mice at 0, 30, 60, and 120 min after glucose administration to measure the blood glucose concentration using a blood glucose meter (Sanuo Biological Sensing Co., Ltd.). The experimental flow was shown in Supplementary Figure 1.

### Body Composition Analysis

Eight mice were randomly selected at the end of the intervention to determine the total fat mass using the body composition analyzer EchoMRI-100H (Huijia Biological Co., Ltd., (China) Co., China).

### Monitoring of Animal Metabolism

At the end of the intervention, four mice were randomly selected from each group to be monitored using the TES PhenoMaster (12-channel) system for 4 days to measure the respiratory entropy metabolism and voluntary movement of mice (24). Each mouse was caged independently, and the ND group was fed a normal diet, whereas the other groups were fed a high-fat diet and could eat and drink *ad libitum*. The first 48 h was considered the adaptation period, and the data obtained were discarded. After that, the stable metabolic monitoring commenced at 14:00 and ended at 14:00 the next day; data point was recorded every 5 min for each mouse. The respiratory exchange ratio (RER) and energy expenditure (EE_1_) were calculated based on the O_2_ consumption and CO_2_ production measured in the exhaust gas of each cage. Body weight was used to correct EE_1_ and obtain EE_2_, and the lean body mass was used to correct EE_2_ and obtain EE_3_. EE and RER values were the direct output by the instrument, and the detailed progress of how to obtain EE and RER is in Supplementary Material 2 Metabolic Software Calculations Template.

### Tissue Sampling and Index Testing

At the end of the intervention period, the mice were fasted for 12 h. Eyeballs were removed for blood collection after inhalation anesthesia with 4% isoflurane (Shenzhen Reward Life Technology Co., Ltd), then euthanized by rapid vertebral dislocation. Body fat (peritesticular and perirenal fat) and liver tissues were collected rapidly, rinsed with normal saline, drained, and weighed. The blood was centrifuged at 2,000 rpm (382 *g*) at 4°C for 15 min, and the supernatant was collected to measure biochemical indicators. All samples were frozen at −80°C for later use.

#### Lipid Body Ratio


$$\begin{array}{c} Lipidbodyratio\left(\%\right)=[Peritesticularfatmass\left(g\right)\\ \;+Perirenalfatmass\left(g\right)]\\ \;÷Bodymass\left(g\right)\times 100\%\left(25\right). \end{array}$$

#### Blood Lipids

Serum was collected using an automatic biochemical analyzer (Minray BS-220) and supporting kits to detect total cholesterol (TC), triglycerides (TG), high density lipoprotein (HLDL), and Low-density lipoprotein (VLDL).

#### Serum Insulin, Leptin, Adiponectin, IL-1β, and TNF-α

Serum was taken to measure the INS, LEP, ADP, IL-1β, and TNF-α levels in mice using mouse enzyme-linked immunosorbent assay (ELISA) kits (Shanghai Sanyan Biotechnology Center), according to the manufacturer’s instructions. The insulin resistance index (HOMA-IR) and islet β-cell function (HOMA-β) were calculated using the following formula (26, 27).


$$HOMA-IR=\frac{INS\left(mUI/l\right)\times FPG\left(mmol/l\right)}{22.51}$$

$$HOMA-\beta =\frac{20\times INS\left(mUI/l\right)}{PFG\left(mmol/l\right)-3.5}$$

#### Hepatic Glucose and Lipid Metabolism-Related Factors

Live tissue was homogenized by adding an appropriate amount of normal saline, centrifuged at 3,000 rpm for 10 min, and the supernatant was collected to measure the expression of lipoprotein lipase (LPL), fatty acid synthase (FAS), sterol regulatory element-binding transcription factor (SREBP-1c), and cholesterol 7α-hydroxylase (CYP7A1) protein in the liver tissue by ELISA, according to the manufacturer’s instructions (Shanghai Sanyan Biotechnology Center).

### 16S rDNA Sequencing

Murine fecal samples were collected 1 day before the test diet intervention and 1 day after the intervention and stored at −80°C. HiPure Stool DNA Kits (Magen, Guangzhou, China) were used to extract total DNA from these samples, after which the conserved 16S rDNA region (V3: 341F, CCTACGGGNGGCWGCAG; V4: 806F, GGACTACHVGGGTATCTAAT) was amplified by PCR (94°C for 2 min; 30 cycles of 98°C for 10 s, 62–66°C for 30 s [55°C for 30 s for 16S V4], 68°C for 30 s, and 68°C for 5 min) using appropriate primers and barcodes. The resultant amplicons were extracted using 2% agarose gel electrophoresis and were purified with AMPure XP Beads (Beckman Agencourt, United States) based on provided directions, followed by quantification with an ABI Step One Plus Real-Time PCR System. Equimolar amounts of these amplicons were pooled and subjected to paired-end sequencing (PE250) on an Illumina instrument (Life Technologies, CA, United States) using standard protocols. Raw reads were filtered to remove low-quality reads, assembled, and filtered using FASTP (v. 0.18.0). Clean tags were clustered into operational taxonomic units (OTUs) at a ≥97% similarity threshold using the UPARSE pipeline (v. 9.2.64). The UCHIME algorithm was employed to remove chimeric tags, and the remaining tags were subjected to downstream analyses. The most abundant sequence within each OTU was selected as a representative sequence. Following OTU determination, gut microbiota, including community composition, alpha diversity, beta diversity, and indicator species, were analyzed. A real-time online Omicsmart platform (Gene Denovo Biotechnology Co., Ltd., Guangzhou, China^1^) was used for these analyses. Taxonomical abundance statistics were visualized using Krona (v 2.6), and dominant bacteria at the phylum and genus levels were analyzed with R. Species comparisons among groups were performed using The Tukey’s HSD test and the Kruskal-Wallis *H* test using the Vegan R package (v 2.5.3) was used to compare species among groups. QIIME (v. 1.9.1, University of Colorado, Boulder, CO, United States) was used to assess changes in the overall microbiota community composition in the ND, HFD, Group 1 (CSE166.67 mg/d.kg), Group 2 (BPL1 1.67×10^9^*cfu*/d.kg), and Group 3 (CSE 166.67 mg/d.kg+BPL1 1.67×10^9^*cfu*/d.*kg*) groups. Principal coordinate analysis (PCoA) was conducted using the R Vegan package (v 2.5.3) and plotted using the ggplot2 package (v. 2.2.1).

### Hematoxylin and Eosin Staining of Liver Tissue

The liver tissue samples were fixed using 4% paraformaldehyde, for more than 24 h. The tissue was placed in the basket in the dehydrator in a gradient alcohol for dehydration: 75% alcohol 4 h – 85% alcohol 2 h – 90% alcohol 2 h – 95% alcohol 1 h – anhydrous ethanol I 30 min – anhydrous ethanol II 30 min – alcohol benzene 5–10 min – xylene I 5–10 min – xylene II 5–10 min – wax I 1 h – wax II 1 h – wax III 1 h. After the tissue was embedded in wax, it was cut into paraffin sections with a thickness of 4 μm with a microtome (RM2016, Shanghai Leica Instruments Co., Ltd). Before using the prepared tissue sections for hematoxylin and eosin HE staining, deparaffinize them to water: sections were sequentially washed in xylene I20 min – xylene 20 min – absolute ethanol I10 min – anhydrous ethanol 10 min – 95% alcohol 5 min – 90% alcohol 5 min – 80% alcohol 5 min – 70% alcohol 5 min – distilled water. Then sections were stained with HE: Harris hematoxylin for 3–8 min – distilled water – stained with eosin dye for 1–3 min – distilled water, Finally, dehydration seal: Sections were sequentially dehydrated in 95% alcohol I 5 min – 95% alcohol II 5 min – absolute ethanol I5 min – absolute ethanol 5 min – xylene I5 min – xylene for 5 min, sections were removed from xylene to slightly dry and neutral gum sealed. Live tissue sections were analyzed by microscopy. paraffin-embedded, cut into sections, stained with hematoxylin and eosin (HE); inflammation and ulceration were then assessed *via* microscopy (Leica-DM4B, Germany).

### Statistical Analysis

Data are expressed as mean, median, standard deviation, and percentiles. Repeated measures data were analyzed for variance using a multivariate variance. If the data distribution was normal and the variance was homogeneous, a one-way analysis of variance (one-way ANOVA) was used to compare the data between groups of one-way measurement indicators, and Fisher’s least significant difference method (LSD) was used for pairwise comparisons between groups. If the data was normal but the variance was no- homogeneous, Dunnett’s T3 was used for pairwise comparisons between groups. The Non-parametric Kruskal–Wallis *H* test was used for comparison between groups for non-normally distributed data, and the Nemenyi method was used for pairwise comparison of the overall mean between groups. Hypothesis test level a = 0.05.

## Results

### Feed Intake and Body Weight

**FIGURE 1:**
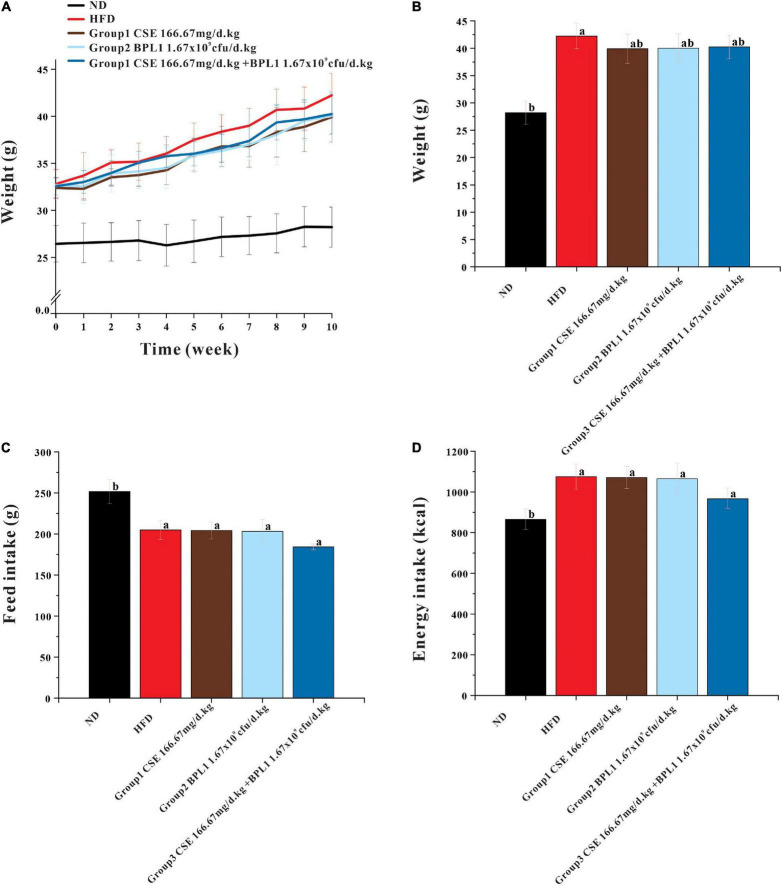
Results of body weight and feed intake. **(A)** Body weight during intervention. **(B)** End-of-intervention body weight in each group. **(C)** Total feed intake. **(D)** Total energy intake. Data are means ± SD (*n* = 12). a: *P* < 0.05, compared with ND. b: *P* < 0.05, compared with HFD.

As showed in Figure 1, at the beginning of the intervention (0 weeks), the average body weights of the HFD group and ND groups were (32.82 ± 1.53) g and (26.45 ± 1.94) g, respectively, the difference was significant (*P* < 0.001). However, no significant difference was observed in the initial body weight of the intervention groups (*P* > 0.05), and the regrouping was reasonable. At the end of the experiments, the body weight of each intervention group was lower than that of the model control group (*P* < 0.05). The body weight of each intervention group was similar, and the difference was not significant (*P* > 0.05). There was no significant difference in the average total feed and energy intake per mouse between the intervention and HFD groups (*P* > 0.05).

### Fat Distribution

**FIGURE 2:**
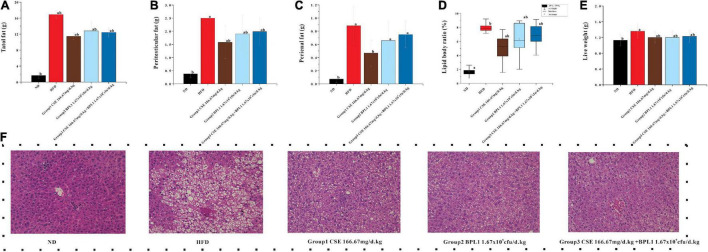
Results of body fat distribution. **(A)** Total body fat weight. **(B)** Peritesticular fat. **(C)** Perirenal fat. **(D)** Lipid body ratio. **(E)** Live weight. **(F)** HE staining of liver tissue. Data are means ± SD (*n* = 12). a: *P* < 0.05, compared with ND. b: *P* < 0.05, compared with HFD.

The total fat, epididymal fat, and perirenal fat of the mice in the HFD group were 10.19, 3.16, and 3.37 times that of mice in the ND group, respectively, and total fats and liver weights in each intervention group were lower than those in the HFD group (*P* < 0.05) after the intervention. Many fat vacuoles appeared, indicating that the high-fat diet increased body fat in mice and fatty liver occurrence. The study suggests that interventions can reduce body fat in mice from a high-fat diet, reduce the damage of visceral fat accumulation in the liver tissue, and inhibit fatty liver formation. As shown in Figure 2, the total fat content of each intervention group was similar (*P* > 0.05), but the effects of different intervention groups on different fat parts were inconsistent. The perirenal fat and epididymal fat of mice in Group 1 (CSE 166.67 mg/d.kg) were lower than those in Group 2 (BPL1 1.67×10^9^*cfu*/d.*kg*), Group 3 (CSE 166.67 mg/d+BPL1 1.67×10^9^*cfu*/d.*kg*), but Group 1 (The liver fat of mice in the CSE 166.67 mg/d.kg) was higher than that in Group 2 (BPL1 1.67×10^9^*cfu*/d.*kg*), and Group 3 (CSE 166.67 mg/d.kg +BPL1 1.67×10^9^*cfu*/d.*kg*) in liver HE slices, and the liver tissue structure recovery was also relatively poor; Group 3 (CSE 166.67 mg/d.kg +BPL1 1.67×10^9^*cfu*/d.*kg*) had the least and smallest hepatic fat vacuoles, and the liver tissue structure was the most similar to the ND group. It is speculated that the reduction of subcutaneous fat by CSE is stronger than that of BPL1, and the reduction of visceral fat by BPL1 is stronger than that of CSE. The combination of CSE and BPL1 can more effectively reduce the accumulation of visceral fat and restore the structure of the liver.

### Blood Lipids Levels

**FIGURE 3:**
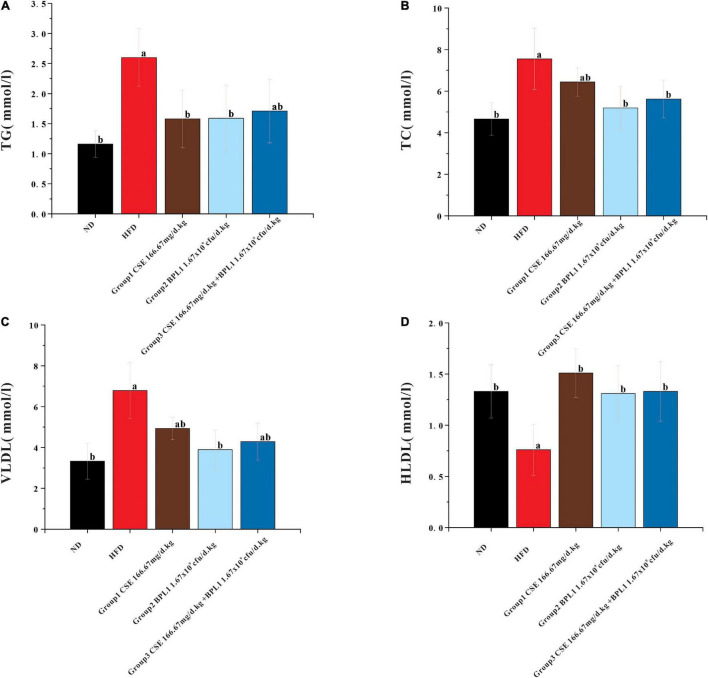
Results of blood lipids levels. **(A)** TG (triglycerides). **(B)** TC (total cholesterol). **(C)** VLDL (very low-density lipoprotein). **(D)** HLDL (high density lipoprotein). Data are means ± SD (*n* = 12); a: *P* < 0.05, compared with ND. b: *P* < 0.05, compared with HFD.

As shown in Figure 3, high-fat diet increased serum TC, TG, and VLDL, but decreased HLDL. While the interventions of the test substance decreased TG, TC, and VLDL, but increased HLDL in obese mice. It shows that the intervention of each test substance can change the dyslipidemia state of the experimental mice caused by a high-fat diet. Using CSE alone to increase HLDL was better than BPL1 alone, but the difference was not significant (*P* > 0.05), CSE + BPL1 alone, and the effect of using BPL1 alone, CSE + BPL1 in reducing TC and VLDL was better than using CSE alone, but the difference was not significant (*P* > 0.05).

### Fasting Blood Glucose Level

**FIGURE 4:**
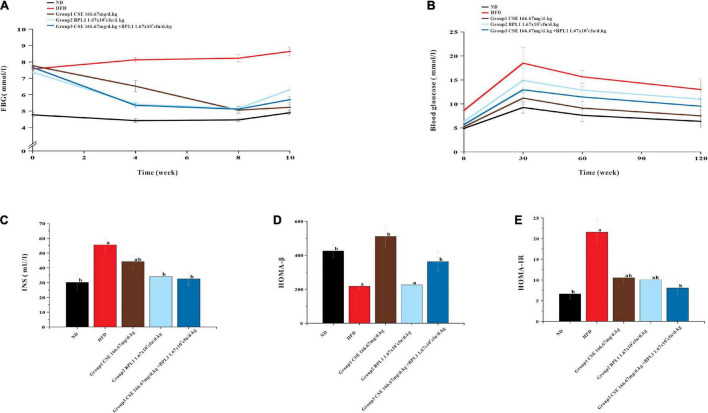
Results of markers of glucose metabolism. **(A)** Fasting blood glucose. **(B)** OGTT (Oral glucose tolerance tests). **(C)** Serum INS (insulin) level. **(D)** HOMA-β (insulin β-cell function). **(E)** HOMA-IR (insulin resistance index). Data are means ± SD (*n* = 12); a: *P* < 0.05, compared with ND. b: *P* < 0.05, compared with HFD.

As shown in Figure 4, the blood glucose of the HFD group showed a slow upward trend, and the blood sugar of each intervention group showed a downward trend. From the 4th week of intervention, the blood glucose of each intervention group was lower than that of the HFD group (*P* < 0.05), indicating that each intervention group could effectively control the increase in fasting blood glucose caused by high-fat diet at the 4th week. From the 4th week of the intervention, the blood sugar of the experimental animals in the combined intervention group was lower than that in the single-use Coix seed intervention group (*P* < 0.05), indicating that the combined use of CSE and BPL1 can quickly show the effect of controlling blood sugar. At the end of the intervention, the experimental animals treated with CSE alone had the best effect on fasting blood sugar control.

### Oral Glucose Tolerance Tests Test

As shown in Figure 4, the area under the curve (AUC) of each intervention group was smaller than that of the HFD group, indicating that each test substance and the combined intervention improved the glycemic ability of the experimental animals. The AUC area of CSE combined with BPL1 was lower than that of BPL1, indicating that the combined intervention improved the blood glucose regulation ability of experimental animals to a greater extent than BPL1 alone. The experimental animals treated with CSE alone had the best blood sugar regulation ability among the intervention groups.

### Serum INS, HOMA-IR, and HOMA-β Levels

As shown in Figure 4, the INS and HOMA-IR levels in each intervention group were lower than those in the HFD group (*P* < 0.05), and HOMA-β was higher than that of in the HFD group (*P* < 0.05), and islet cell function improvement was similar to that in the ND group. Moreover, INS, HOMA-β and HOMA-IR levels in Group 1 (CSE 166.67 mg/d.kg) were higher than other groups (*P* < 0.05). It shows that the high-fat diet has certain damage to the islet cell function of the experimental mice, and the mice in the HFD group may develop insulin resistance. Each test substance and combined intervention can reduce the damage to the experimental mice’s islet function caused by a high-fat feeding, and protect the islet function and pancreatic β-cell functions. The experimental animals treated with CSE alone showed the strongest islet ability.

### Indicators Related to Energy Metabolism

**FIGURE 5:**
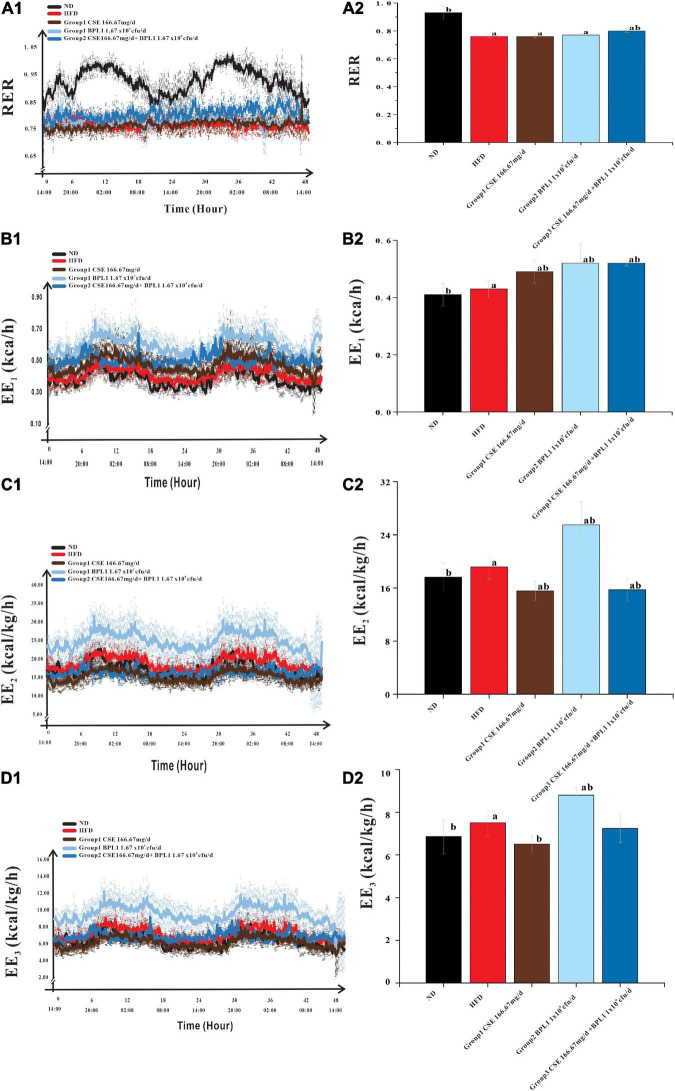
Energy Metabolism-Related Indicators. **(A1)** Change in the respiratory exchange ratio (RER) over time. **(A2)** Comparison of the mean RER of each group. **(B1)** Change in the energy expenditure (EE_1_) over time. **(B2)** Comparison of the mean EE_1_ of each group. **(C1)** Change in the energy expenditure (EE_2_) over time. **(C2)** Comparison of the mean EE_2_ of each group. **(D1)** Change in the energy expenditure (EE_3_) over time. **(D2)** Comparison of the mean EE_3_ of each group. The body weight was used to correct EE_1_ to obtain EE_2_, and the lean body mass was used to correct EE_2_ to obtain EE_3_. Data are means ± SD (*n* = 12); a: *P* < 0.05, compared with ND. b: *P* < 0.05, compared with HFD.

As shown in Figure 5, the change in RER over time of the HFD group remained stable at around 0.76, while that of the ND group had a clear circadian rhythm, and was close to 1.00 at night, indicating the energy consumption substrate of the model obese mice mainly comes from fat, and glucose is less used as the energy consumption substrate; after the intervention of the test substance, the RER of the obese mice increases, indicating that the intervention groups reduce the proportion of fat consumption of the obese mice. The proportion of fat in the substrate increases the proportion of glucose in the metabolic substrate. The RER of CSE combined with BPL1 was higher than that of CSE or BPL1 alone (*P* < 0.05), indicating that the combined use of CSE and BPL1 has a synergistic effect, increasing the proportion of glucose in the metabolic substrate.

Energy consumption of the body consists of three parts: resting energy consumption, food thermogenic effect, and activity energy consumption. As shown in Table 2, the EE_1_, EE_2_, and EE_3_ of the HFD group were higher than those in the ND group (*P* < 0.05). However, the amount of exercise performed was lower than that in the ND group (*P* < 0.05).

**TABLE 2:** Result of energy metabolism-related indicators.

Group	Test substance	Dose (mg/d.kg or cfu/d.kg)	RER ($\bar{X}\pm S$)	EE_1_ ($\bar{X}\pm S$,kcal/h)	EE_2_ ($\bar{X}\pm S$,kcal/kg/h)	EE_3_ ($\bar{X}\pm S$,kcal/kg/h)	Amount of exercise (m/day)
ND	Normal saline	–	0.93 ± 0.05*^b^*	0.41 ± 0.04*^b^*	17.62 ± 2.18*^b^*	6.86 ± 0.85*^b^*	1299.19 ± 308.01*^b^*
HFD	Normal saline	–	0.76 ± 0.01*^a^*	0.43 ± 0.03*^a^*	19.16 ± 1.79*^a^*	7.51 ± 0.74*^a^*	713.00 ± 133.28*^a^*
Group 1	CSE	166.67	0.76 ± 0.01*^a^*	0.49 ± 0.04*^ab^*	15.57 ± 1.45*^ab^*	6.51 ± 0.60*^b^*	1180.00 ± 146.19*^b^*
Group 2	BPL1	1.67×10^9^	0.77 ± 0.01*^a^*	0.52 ± 0.07*^ab^*	25.50 ± 3.49*^ab^*	8.80 ± 0.34*^ab^*	1541.03 ± 223.71*^b^*
Group 3	CSE+BPL1	166.67+1.67×10^9^	0.80 ± 0.01*^ab^*	0.52 ± 0.01*^ab^*	15.73 ± 1.73*^ab^*	7.24 ± 0.90	1171.27 ± 149.60*^b^*

*^a^P < 0.05, compared with ND. ^b^P < 0.05, compared with HFD.*

This shows that because the body size of obese mice is larger than that of normal mice, and the food intake is a high-fat diet, even if the active energy consumption is low, the resting energy consumption and the thermogenic effect of food are higher, which contributes more to the total energy consumption of obese mice. After the intervention of the test substance, the exercise volume of the obese mice was higher than that of the HFD group (*P* < 0.05), indicating that the intervention of the test substance could increase the activity energy consumption value by increasing the exercise volume.

However, the changes in energy consumption in different intervention groups were inconsistent. The EE_1_ of the mice in the unadjusted weight-adjusted group was higher than that in the HFD group (*P* < 0.05), but the EE_2_ and EE_3_ of the mice in the adjusted weight and lean body mass were lower than HFD group (*P* < 0.05). Combined Group 1 (CSE 166.67 mg/d.kg) and Group 3 (CSE 166.67 mg/d+BPL1 1.67×10^9^*cfu*/d.*kg*) mice weighed less than the HFD group (*P* < 0.05), feed intake were similar to HFD group (*P* > 0.05), and the amount of exercise was higher than the HFD group, suggesting that CSE or CSE + BPL1 can improve the exercise volume and increase the value of active energy consumption in obese mice, but the changes in resting energy consumption and the thermogenic effect of food are not obvious, resulting in higher energy consumption value in the HFD group after adjusting for body weight and lean body mass. Combined Group 2 (BPL1 1.67×10^9^*cfu*/d.*kg*) mice weighed less than the HFD group, feed intake was similar to HFD group (*P* < 0.05), and the amount of exercise was higher than the HFD group (*P* < 0.05), suggesting that the BPL1 not only increases the value of active energy expenditure, but also increases resting total energy expenditure and or the thermogenic effect of food. The energy expenditure value of the HFD group was still lower, even after adjusting for body weight and lean body mass.

### Serum IL-1β, TNF-α, Leptin and Adiponectin Levels

**FIGURE 6:**
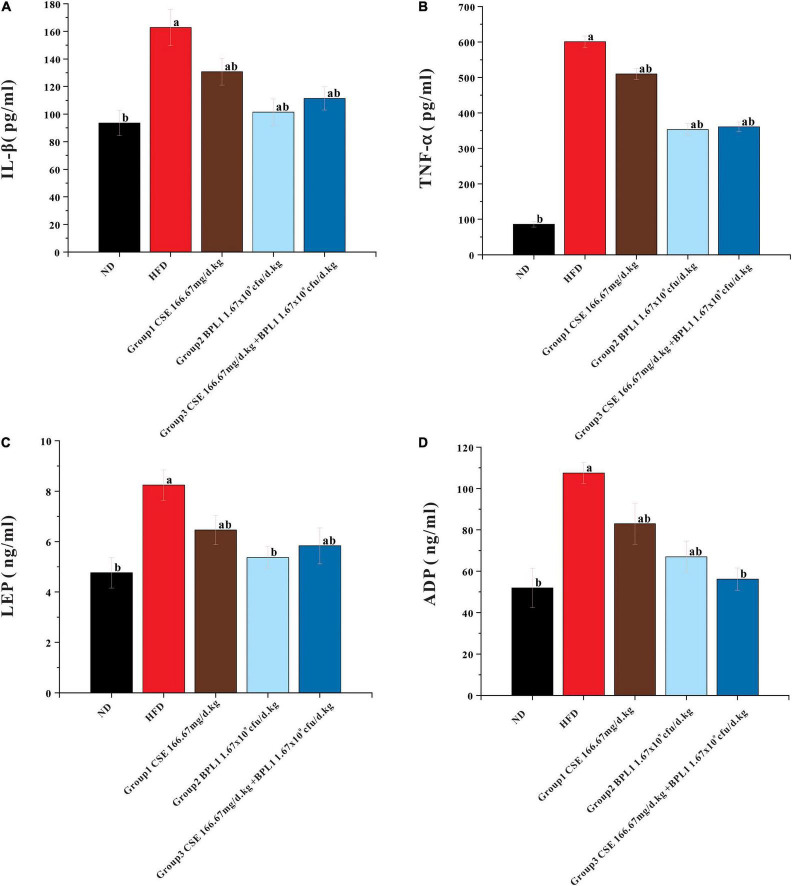
Results of serum glycolipid metabolism-related indicators. **(A)** Inflammatory factors IL-1β. **(B)** Inflammatory factors TNF-α. **(C)** Serum LEP (Leptin) level. **(D)** Serum ADP (Adiponectin) level. Data are means ± SD (*n* = 12); a: *P* < 0.05, compared with ND. b: *P* < 0.05, compared with HFD.

As shown in Figure 6, serum IL-1β and TNF-α levels in each intervention group were lower than those in the HFD group (*P* < 0.05). The serum levels of IL-1β and TNF-α in Group 2 (BPL1 1.67×10^9^*cfu*/d.*kg*) and Group 3 (CSE 166.67 mg/d+BPL1 1.67×10^9^*cfu*/d.*kg*) were lower than those in Group 1 (CSE 166.67 mg/d.kg) (*P* < 0.05). Each intervention group can reduce the levels of serum IL-1β and TNF-α. The effect of BPL1 alone and CSE + BPL1 in reducing inflammation is stronger than that of CSE alone (*P* <0.05).

The serum LEP and ADP levels in each intervention group were lower than those in HFD group (*P* < 0.05). LEP and ADP serum levels in Group 2 (BPL1 1.67×10^9^*cfu*/d.*kg*) and Group 3 (CSE 166.67 mg/d+BPL1 1.67×10^9^*cfu*/d.*kg*) were lower than those in Group 1 (CSE 166.67 mg/d.kg) (*P* < 0.05). The effect of BPL1 alone and CSE+BPL1 interventions in reducing LEP and ADP were stronger than those of CSE alone.

### Lipoprotein Lipase, CYP7A1, Fatty Acid Synthase and SRBBP-1c Levels in Liver Tissue

**FIGURE 7:**
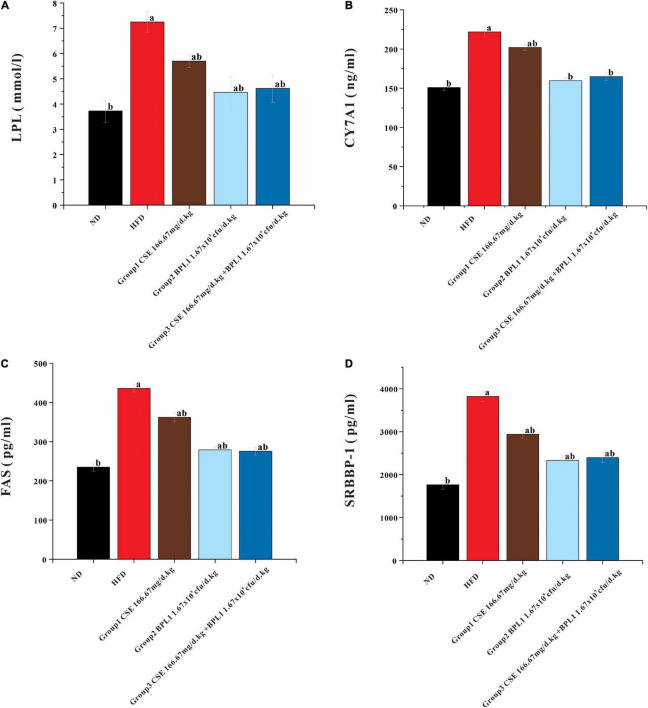
Results of glycolipid metabolism-related indicators in liver tissue. **(A)** LPL (lipoprotein lipase). **(B)** FAS (fatty acid synthase). **(C)** CYP7A1 (cholesterol 7α-hydroxylase). **(D)** SREBP-1 (Sterol regulatory element binding transcription factor-1). Data are means ± SD (*n* = 12). a: *P* < 0.05, compared with ND. b: *P* < 0.05, compared with HFD.

As shown in Figure 7, the LPL, CYP7A1, FAS, and SRBBP-1 levels in the liver tissue of each intervention group were lower than those of the HFD group (*P* < 0.05). LPL, CYP7A1, FAS, and SRBBP-1c levels in the liver tissues of Group 2 (BPL1 1.67×10^9^*cfu*/d.*kg*) and Group 3 (CSE 166.67 mg/d+BPL1 1.67×10^9^*cfu*/d.*kg*) were lower than those in Group 1 (CSE 166.67 mg/d.kg) (*P* < 0.05). BPL1 alone and CSE+BPL1 interventions in reducing liver tissue glucose and lipid metabolism-related protein were stronger than that of CSE alone.

### Gut Microbiome Results

#### Species Composition Analysis

**FIGURE 8:**
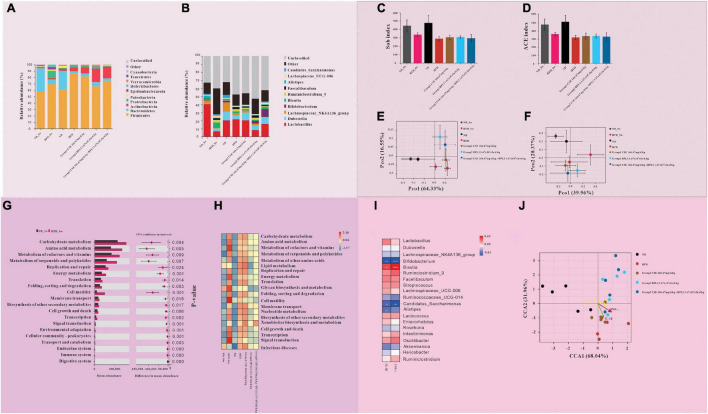
Results of gut microbiome. **(A,B)** Gut microbiota composition at the **(A)** phylum and **(B)** genus levels. **(C–F)** Intestinal flora diversity analysis. **(C)** Sob index and **(D)** ACE index of α diversity of intestinal flora. **(E)** β diversity of intestinal flora at the phylum levels. **(F)** β diversity of intestinal flora at the genus levels. **(G,H)** Gut microbiota functional analysis. **(G)** Comparison of microbiota function in obese and normal mice at the initiation of intervention. **(H)** Comparison of microflora function of each group at the end of the intervention. **(I,J)** The relationship between inflammatory factors and intestinal flora. **(I)** Correlation between gut microbiota and inflammatory factors. **(J)** The relationship between different groups of intestinal flora and inflammatory factors.

Figures 8A,B, shows the results of the species composition of the intestinal flora. The intestinal flora of the obese mice at the beginning of the intervention differs from that of the normal mice at the phylum and the genus level. (1) At the phylum level, the proportion of Firmicutes, Proteobacteria, Actinobacteria, and Tenericutes in obese mice were greatly increased, While Bacteroidetes were greatly decreased. (2) At the genus level, Lactobacillus and Lachnospiaceae_NK4A136_group decreased greatly, and Blautia increased in obese mice.

At the end of the intervention, the intestinal microbiota structure of mice in each intervention group, the HFD group, and the initial obese mice was different. (1) At the phylum level, compared with HFD_0w, each test substance group and HFD group decreased the types of flora, increased the proportions of Firmicutes and Actinobacteria, and decreased the proportions of Proteobacteria and Bacteroidetes. Compared with the HFD group, the proportions of Firmicutes and Proteobacteria in intervention groups decreased, while the proportions of Bacteroidete and Actinobacteria increased. Among the intervention groups, Bacteroidete and Actinobacteria increased the most in Group 2 (BPL1 1.67×10^9^*cfu*/d.*kg*), and Firmicutes decreased the most, followed by Group 3 (CSE 166.67 mg/d+BPL1 1.67×10^9^*cfu*/d.*kg*) and finally Group 1 (CSE 166.67 mg/d.kg). (2) At the genus level, compared with HFD_0w, Lactobacillus and Dubosiella increased and Blautia decreased in intervention groups and HFD group. Compared with the HFD group, Group 1 (CSE 166.67 mg/d.kg) and HFD group had basically similar flora structures, while Group 2 (BPL1 1.67×10^9^*cfu*/d.*kg*) and Group 3 (CSE 166.67 mg/d+BPL1 1.67×10^9^*cfu*/d.*kg*) had more species, Lactobacillus decreased, and Bifidobacterium increased.

#### Species Diversity Comparison

Figures 8C,D shows the α species diversity comparison. Before the intervention, the species richness of obese mice was lower than that of normal mice. However, the species richness of the HFD group decreased further than before and at the end of the intervention, and the species richness of the normal mice did not change before and after the intervention. Compared with the HFD group, the species richness of intervention groups increased. Figures 8E,F shows β s species diversity comparison. Before and after the intervention, regardless of the phylum or genus level; the flora composition of normal mice was similar, and the flora difference of obese mice before and after the intervention was larger than that of normal mice. The results of the principal component analysis of the microbiota of Group 1 (CSE 166.67 mg/d.kg) mice were similar to those of the HFD group, while that of Group 2 (BPL1 1.67 10^9^*cfu*/d.*kg*) and Group 3 (CSE 166.67 mg/d+BPL1 1.67×10^9^*cfu*/d.*kg*) were significantly different from those of the HFD group.

#### Bacterial Function Comparison

As shown in Figures 8G,H, there were significant differences in 20 pathways between obese mice and normal mice at the beginning of the intervention. In particular the pathways of carbohydrate metabolism, amino acid metabolism, and energy metabolisms were more abundant in obese mice. After 10 weeks of intervention, the abundance of pathways involved in glucose metabolism and energy metabolism in the intestinal flora of mice in the ND group did not change significantly, Whereas the abundance of pathways involved in lipid metabolism increased. The abundance of pathways involved in glucose metabolism and energy metabolism in the intestinal flora of mice in the HFD group decreased, while those involved in lipid metabolism increased. Group 1 (CSE 166.67 mg/d.kg) and HFD groups had similar microbial functions, but Group 2 (BPL1 1.67×10^9^*cfu*/d.*kg*), Group 3 (CSE 166.67 mg/d+BPL1 1.67×10^9^*cfu*/d.*kg*) and HFD groups had significantly different microbial functions. The abundance of pathways involved in glucose, lipid, and energy metabolisms was significantly reduced compared with the HFD group.

#### Relationship Between Inflammatory Factors, Flora and Groups

The abundance of Bifidobacterium, Candidatus_Saccharimonas, and Alistipes were inversely proportional to the concentration of inflammatory factors (IL-1β and TNF-α), and the abundance of Blautia was proportional to the concentration of inflammatory factors (IL-1β, TNF-α) Figures 8I,J. The species in the HFD group, the Group 1 (CSE 166.67 mg/d.kg) had a large intersection and a short distance and had a strong positive correlation with inflammatory factors (IL-1β and TNF-α). However, the intersection of Groups 2, 3, and HFD was small, the distance was laeger, and the correlation with inflammatory factors (IL-1β and TNF-α) was weak.

## Discussion

Obesity is caused by an imbalance between energy intake and consumption. Excessive nutrients are ingested, but energy expenditure does not increase. Cells or organs convert the excess nutrients into fat for storage and accumulate in the subcutaneous and internal organs. This, in turn, affects the normal organ functions, resulting in glucose and lipid metabolism disorders and the development of metabolic-related chronic diseases (28). This study found that the use of CSE, BPL1 and a combination of CSE and BPL1 could improve the energy metabolism of obese mice, control the weight gain in obese mice, and reduce lipid accumulation, and regulate abnormal blood lipids and blood sugar profiles without changing feed intake. There were no significant differences in final body weight between different intervention groups. However, this study found the different effects of CSE and BPL1 and their combination on intestinal flora regulation, inflammatory state, fat distribution, energy metabolism and glucose, and lipid metabolism in obese mice. There is a need to further discuss the related mechanisms regulating the efficacy of CSE, BPL1 and their combination which would help prevent and control obesity and obesity-related diseases.

### Different Pathways Reduce Inflammation Levels and Affect Glucose and Lipid Metabolism

This study found that the RER of obese mice in each intervention group increased, indicating that the ratio of glucose in the energy metabolism substrate was increased, which may be the test substance intervention to reduce the level of serum inflammatory factors (IL-1β and TNF-α), relieve insulin resistance, and improve the body’s ability to utilize sugar (29, 30). A chronic low-grade inflammatory state is a major factor in the progression of chronic metabolic disease (31, 32). Obesity increases endoplasmic reticulum stress, leading to the unfolded protein response activation, which triggers NF-kb, JNK, and other pathways and increases oxidative stress, leading to the upregulation of inflammatory cytokines (33). Simultaneously, obesity causes hypertrophy of adipocytes in white adipose tissue, increases free fatty acids, changes adipokines, induces inflammatory responses and affects glucose and lipid metabolism (32–35). Therefore, improving the inflammatory state of obesity and the abnormal glucose and lipid metabolism may delay the progression of chronic STDs progression. IL-1β can promote inflammatory cytokines release by mediating islet β-cell apoptosis or activating the NF-κB pathway, thereby inducing insulin resistance and type 2 diabetes (36, 37). TNF-α can promote the release of free fatty acids (FFA) through the hormone-sensitive lipase (HSL) pathway, which can increase TNF-α secretion from macrophages through the TLR4/NF-κB pathway, resulting in a vicious circle of mutual promotion of TNF-α and FFA. TNF-α can participate in insulin resistance-induced brown adipocyte apoptosis and atrophy, and promote the formation of obesity with hyperinsulinemia (36, 38, 39), and promote the progression of insulin resistance.

This study showed that the effects of the different test substance on inflammation and blood sugar regulation differed. Although CSE reduced the levels of IL-1β and TNF-α, the effect was not as strong as that of BPL1 alone or CSE+ BPL1. Although the inflammation levels of BPL1 and CSE + BPL1 were lower than that of CSE, the blood sugar regulatory effect was not as strong as that of CSE alone. We speculating the reasons: BPL1 alone and CSE + BPL1 could play a role in regulating the intestinal flora in a chronic inflammatory state, but no changes in the abundance of bacteria that could reduce the level of inflammatory factors were found after CSE intervention. Studies have shown that obesity, intestinal flora dysfunction, and chronic low-grade inflammation are inextricably associated. Disturbed gut flora can alter the function of the gut flora, thereby increasing the absorption and storage of energy substances, leading to obesity, and it can also change the metabolites of gut flora, promoting the production of chronic low-grade inflammation and leading to obesity. In the analysis of intestinal flora, it was found that the structure of intestinal flora in the BPL1 or CSE + BPL1 intervention groups was changed, showing an increase in the proportion of Bifidobacterium and a decrease in the proportion of Lactococcus. Studies have shown that Lactococcus has been reported as a taxon associated with obesity (40). Its abundance is positively correlated with the levels of pro-inflammatory cytokines, such as IL-1β and TNF-α (41). In this study, it was also found that the increase in Bifidobacterium was negatively correlated with the levels of inflammatory factors IL-1β and TNF-α, and the increase in Lactococcus was positively correlated with the levels of inflammatory factors IL-1β and TNF-α. Therefore, it is speculated that BPL1 or CSE + BPL1 can increase the abundance of Bifidobacterium and reduce the abundance of Lactococcus by changing the structure of the intestinal flora, thereby improving the level of inflammatory factors.

CSE was not found to significantly change the abundance of bacteria with anti-inflammatory effect. It is speculated that it is more likely to be absorbed into the blood by its components in the intestine, directly synthesizing inflammatory factors in the body and affecting the related factors of fat synthesis. According to the results of ADP and LEP levels in the serum of the mice in the CSE intervention group, it could be speculated that the CSE can exert its effect by regulating the adiponectin–adenosine monophosphate-activated protein kinase–acetyl-CoA carboxylase–malonyl-Coenzyme A –free fatty acid lipid metabolism pathway, reducing the content of free fatty acids in the blood (42), increasing the content and activity of antioxidant enzymes, reducing the content of serum free radicals (43, 44). CSE also regulates neuroendocrine activity in the brain, reducing LEP synthesis in obese individuals, and controlling energy balance (45, 46). Studies have shown that in obese individuals, reducing fatty acid content, free radical content, and LEP level and increasing antioxidant enzyme activity can help reduce the level of inflammatory factors (36, 38, 39).

CSE has a protective effect on intestinal mucosal immunity, helps to repair the intestinal barrier, and promotes the transmission of hypoglycemic signaling pathways. In a study on the protective effect of CSE on colitis mucosa, T cell regulation was one of the targets of CSE (47, 48), reducing the oxidative stress level in the intestine, reducing the secretion of TNF-α in the intestinal mucosa, and protecting the intestinal mucosa (47, 49). Research on blood sugar regulation of type 2 diabetes by CSE found that CSE can reduce colonic mucosal damage, increase serum insulin levels and increase HLDL levels to control blood sugar. This process is related to the IGF1/PI3K/AKT signaling pathway activation by CSE (50). IGF1 is positively correlated with the level of HLDL in diabetic mice, and the increase in serum IGF1 is beneficial for the decrease in blood glucose levels in diabetic mice (51). This study was also found that the serum insulin INS and HLDL levels of CSE alone were higher than those of BPL1, CSE + BPL1.

### Different Pathways Control Energy Balance and Affect Fat Synthesis and Distribution

Each intervention group increased the amount of exercise and energy consumption, reduced the accumulation of fat, and controlled the weight gain of experimental animals. Studies have shown that mice on a long-term high-fat diet show a certain state of depression under high-energy loads, which reduces the activity of obese mice (29, 52, 53). This experiment found that the exercise amount in the HFD group decreased and the blood sugar and blood lipid levels increased. After the intervention with the test substance, the exercise amount and energy metabolism of the obese mice increased, and the blood sugar and blood lipid levels were significantly reduced. This indicates that the intervention of the test substance regulates the metabolic state of glucose and lipids and improves the depression in mice due to hyperglycemia and hyperlipidemia (53). It also increased the amount of exercise in obese mice, improved the level of energy metabolism, reduced the accumulation of lipids, and controlled weight gain.

Each intervention can control weight gain by reducing the chronic inflammatory state and fat synthesis. Studies have shown that a chronic low-grade inflammatory state exacerbates endoplasmic reticulum stress (54, 55), promoting the overexpression of SREBP-1c by regulating the PERK-SREBP1c -FAS signaling pathway (56), thereby causing the overexpression of FAS protein in the body. This promotes lipogenesis, leading to excessive fat accumulation in non-adipose tissue (57–59), promoting metabolic disease progressions, such as obesity, insulin resistance, type 2 diabetes and fatty liver (60–63). Both CSE and BPL1 intervention alone or in combination can reduce the content of these two proteins. It is speculated that the reduction of liver fat weight in the intervention group might be by reducing the level of inflammation and reducing endoplasmic reticulum stress, thereby regulating the PERK-SREBP1c -FAS signaling pathway, and reducing the content of SRBBP-1 protein and FAS protein in the liver tissue, thereby reducing the synthesis and accumulation of fat.

The control of energy balance, lipid synthesis, and distribution of each intervention group were differed. This study found that BPL1 and CSE + BPL1 increased the basal metabolic rate and reduced the fat accumulation in liver tissue better than the CSE alone. This showed in the improvement of EE_2_ (EE adjusted for weight), EE_3_ (EE adjusted for lean body mass), and liver fat in HE slices of liver tissue and decreased liposynthesis protein expression. These results may differ from the process of CSE and BPL1 exerting energy balance control. The study found that the obese microbiota had a higher relative abundance of bacteria capable of fermenting carbohydrates, increased the absorption of monosaccharides in the intestinal lumen, which in turn increased insulin and glucose levels in the body, induced the expression of lipogenic enzymes in the liver, and promoted the lipid biosynthesis and fat accumulation in the host (64, 65). In this study, the analysis of the intestinal flora function of each group also revealed that the species abundance of glucose metabolism, energy metabolism and lipid metabolism in the HFD group was higher. After BPL1 and CSE + BPL1 intervention, the species abundances of glucose, energy and lipid metabolism were significantly reduced. It is speculated that BPL1 and CSE + BPL1 can reduce the absorption of nutrients and then reduce lipid synthesis.

Moreover, gut microbiota can promote LPL expression by inhibiting FIAF gene expression (65, 66), thereby adipocyte triglyceride storage. This study, also found that the BPL1 and CSE + BPL1 intervention groups reduced LPL protein synthesis more strongly than CSE, and it is speculated that the BPL1, CSE + BPL1 intervention groups may inhibit the expression of the host FIAF gene, reduce the protein synthesis of LPL, and then reduce fat accumulation. Studies have shown that increasing the gut microbiota metabolite butyrate increases the phosphorylation of peroxisome proliferator-activated receptor-gamma coactivator-1α (PGC-1α) and AMPK in the liver and muscle and the expression of PGC-1α and mitochondrial uncoupling protein-1 (UCP-1) to promote fatty acid oxidation and thermogenesis (67, 68). In this study, it was found that the EE of the BPL1 group after adjusting the body weight was higher than that of using CSE alone, which may be related to the increase in butyrate, a metabolite of intestinal flora, thereby enhancing metabolic heat production. In terms of reducing liver fat synthesis and increasing energy metabolism, the effects of BPL1 and CSE + BPL1 are stronger than that of CSE alone, which may be related to the intestinal flora.

This study compared the effect characteristics of CSE, BPL1 and their combination on glucose and lipid metabolism in obese mice, and provided the reference for the combined use of plant extracts and probiotics. The combination of CSE + BPL1 designed only one dose group in this study, and required further exploration of the dose-effect relationship. The brown fat samples were not collected, and the effects of the interventions on brown fat need to be further explored.

## Conclusion

CSE, BPL1 and their combination can effectively control the weight gain in obese mice, reduce fat content, and regulate blood lipids and abnormal blood sugar. This may be related to the test substance intervention that can reduce the chronic inflammatory state, improve energy metabolism, and exercise, relieve insulin sensitivity, and reduce lipid synthesis. Different test substances exert their effects in different ways. CSE may directly act on body tissues to exert anti-inflammatory effects, regulate glucose and lipid metabolism through intestinal absorption into the blood, protect the intestinal mucosa, regulate hypoglycemic pathways, and improve insulin resistance. BPL1 and CSE + BPL1 may improve the structure and function of the intestinal flora, reduce tissue inflammation, reduce nutrient absorption, increase energy metabolism, and regulate glucose and lipid metabolism. Compared with the single use of CSE, the combination of CSE + BPL1 can better exert the regulation function of intestinal flora. Compared to BPL1 alone, the combination of CSE + BPL1 can better regulate pancreatic islet function and improve blood sugar.

## Data Availability Statement

The datasets presented in this study can be found in online repositories. The names of the repository/repositories and accession number(s) can be found below: NCBI, Accession: PRJNA854286.

## Ethics Statement

The animal study was reviewed and approved by the Animal Ethical and Welfare Committee of the Laboratory Animal Center of Xiamen University.

## Author Contributions

WZ, HoL, and HF conceived and designed the experiments. WZ, YX, QX, HZ, and XJ performed the experiments. WZ, MZ, and ZZ analyzed the data. WZ, JH, YL, W-HL, XM, WH, and HF contributed reagents, materials, and analysis tools. WZ and HoL wrote the manuscript. All authors contributed to the study conception and design, read, and approved the final manuscript.

## Conflict of Interest

XJ, ZZ, JH, HaL, JD, YL, W-HL, XM, WH, and HF Inner Mongolia Dairy Technology Research Institute Co., Ltd., and Inner Mongolia Yili Industrial Group Co., Ltd. The remaining authors declare that the research was conducted in the absence of any commercial or financial relationships that could be construed as a potential conflict of interest.

## Publisher’s Note

All claims expressed in this article are solely those of the authors and do not necessarily represent those of their affiliated organizations, or those of the publisher, the editors and the reviewers. Any product that may be evaluated in this article, or claim that may be made by its manufacturer, is not guaranteed or endorsed by the publisher.

## Footnotes

1
http://www.omicsmart.com

## Supplementary Material

Supplementary Figure 1 Experimental flow graph.

Supplementary Material 2 Metabolic Software Calculations.xlsx.

Supplementary Material 3 Coix Seed Product Inspection Sheet.pdf.
