# Supplementary material for: Effects of Coix Seed Extract, Bifidobacterium BPL1, and Their Combination on the Glycolipid Metabolism in Obese Mice
Source: Front Nutr. 2022 Jul 18;9:939423. doi: 10.3389/fnut.2022.939423 (PMC9341295; doi:10.3389/fnut.2022.939423)
Supplement: Supplementary Material 3 — Coix Seed Product Inspection Sheet.pdf. [file Data_Sheet_1.PDF]

产品检验报告单

薏苡仁（薏米）提取物　30% 多糖（UV）

产品批号：CYYR-C-A003776　生产日期：20201224　失效日期：20221223

**常规信息**			
使用部位	种子	提取溶剂	水和乙醇
植物来源	*Coix lacryma-jobi L.*	原产地	中国
**检测项目**	**规格标准**	**检测标准**	**检测结果**
**理化数据**			
颜色	灰白色	感官检测	符合规定
气味	特殊气味	感官检测	符合规定
外观	精细粉末	感官检测	符合规定
**产品指标**			
鉴别	与标准样品图谱一致	高效薄层色谱	一致
多糖	≥30.0%	UV	30.11%
粒度	95 % 通过 80 目筛	欧洲药典 <2.9.12>	符合规定
干燥失重	≤5.0 %	欧洲药典 <2.8.17>	1.05 %
灰分	≤10.0 %	欧洲药典 <2.4.16>	1.92 %
松散密度	40~60 g/100mL	欧洲药典 <2.9.34>	41 g/100mL
夯实密度	60~90g/100mL	欧洲药典 <2.9.34>	68 g/100mL
**限量物质**			
铅（Pb）	≤3.0 mg/kg	欧洲药典 <2.2.58>ICP-MS	0.0256 mg/kg
砷（As）	≤2.0 mg/kg	欧洲药典 <2.2.58>ICP-MS	0.1189 mg/kg
镉（Cd）	≤1.0 mg/kg	欧洲药典 <2.2.58>ICP-MS	<0.01 mg/kg
汞 （Hg）	≤0.1 mg/kg	欧洲药典 <2.2.58>ICP-MS	<0.01 mg/kg
**微生物**			
总细菌数	≤10000 cfu/g	欧洲药典 <2.6.12>	10 cfu/g
霉菌&酵母菌	≤1000 cfu/g	欧洲药典 <2.6.12>	10 cfu/g
大肠杆菌	不得检出	欧洲药典 <2.6.13>	符合规定
沙门氏菌	不得检出	欧洲药典 <2.6.13>	符合规定
**包装储存**	硬纸板桶，内衬双层塑料袋；<br>每桶净重25kg，直径35×高51cm；<br>在密封、避光的环境中，常温、干燥的条件下。		
**保质期**	上述条件下可保存24个月。		
**生产商**	**陕西嘉禾生物科技股份有限公司**		

质检负责人　崔鹏

复核人　陕西嘉禾生物科技股份有限公司 质检专用章

检验人　锋惠印战

No：STP-QCP-1（345）
